# Repellent Activity of DEET and Biont-Based Mosquito Repellents in the Chinese Market Against the Asian Long-Horned Tick, *Haemaphysalis longicornis*

**DOI:** 10.3390/insects16050467

**Published:** 2025-04-29

**Authors:** Weiqing Zheng, Yuyang Zhang, Jingzhi Huang, Qinglu Wu, Jintong Fu, Yongwei Wen, Siyu Fang, Xiaoyan Yang, Qianfeng Xia

**Affiliations:** 1 NHC Key Laboratory of Tropical Disease Control, School of Tropical Medicine, Hainan Medical University, Longhua District, Haikou 571199, China; zyuyang2025@163.com (Y.Z.); 18775463709@163.com (J.H.); 18289619461@163.com (Q.W.); fly15979920706@163.com (J.F.); 13198918562@163.com (Y.W.); 18689616235@163.com (S.F.); 15111736779@163.com (X.Y.); 2 Jiang Pest Control (Guangzhou) Co., Ltd., Huadu District, Guangzhou 510880, China

**Keywords:** mosquito repellent, DEET, biont-based repellent, tick, developmental stages

## Abstract

To investigate the repellent efficacy of commercially available mosquito repellents in China against *Haemaphysalis longicornis*, four representative DEET-based repellents and biont-derived repellents were selected. This study utilized a modified circular filter paper method repellent testing device to establish an evaluation system for assessing the repellent effects of each product against three developmental stages of *H. longicornis*: larvae, nymphs, and adults. In our study, for DEET-based repellents, Johnson demonstrated the highest repellency against larvae within 1 h, with an average repellency rate exceeding 80.14%. Yamei and Johnson exhibited repellency rates more than 91.11% for nymphs within 1 h, surpassing the other two DEET products. Repellency rates of Longliqi and Xiaohuanxiong fluctuated between 80.95% and 100% at different time points. Yamei, Longliqi, and Johnson achieved 100% repellency to adults within 1 h, while Xiaohuanxiong showed slightly lower efficacy. The four biont-derived repellents showed significant variations in efficacy: larval-stage repellency ranged from 14.29% to 88.89%, nymphal-stage repellency from 57.89% to 100%, and adult-stage repellency from 50% to 79.49%. CaliforniaBaby exhibited the highest efficacy, comparable or superior to DEET-based products, whereas Longhu demonstrated the weakest repellency and poor persistence. We further conducted a persistence test for CaliforniaBaby and found that it maintained >75% repellency against *H. longicornis* for 6 h. This study provides scientific evidence for selecting tick repellents in practice, offers guidance for purchasing commercial biont-derived tick repellents, and serves as a reference for developing safer, more effective tick repellents.

## Simple Summary

The longhorned tick (*Haemaphysalis longicornis*) is one of China’s most dangerous disease-carrying ticks. It spreads serious illnesses like SFTS virus, putting people at high risk. Right now, chemical pesticides are the main way to control these ticks. But with more people spending time outdoors, personal protection is key. The best method to avoid tick bites is applying repellent to exposed skin. However, China’s market lacks specialized tick repellents, and it’s unclear if common mosquito repellents work against ticks. To test this, we studied 8 popular mosquito repellents sold in China—four with DEET (a common chemical) and four natural biont-derived products known to repel ticks. Our experiments showed most of these mosquito repellents also keep ticks away, especially from nymphs and adult ticks, and the protection lasts a long time. So, if someone needs tick protection, these mosquito repellents in China could be a practical choice while specialized products are unavailable.

## 1. Introduction

In recent years, China has faced escalating public health risks posed by tick-borne diseases, particularly those linked to *Haemaphysalis longicornis* (Asian longhorned tick). This species serves as a vector for severe fever with thrombocytopenia syndrome (SFTS), a life-threatening viral disease with a 12–50% fatality rate [1]. Since its first identification in 2009, SFTS cases have surged, with over 1500 infections reported annually across Henan, Hubei, Shandong, Anhui, Liaoning, Jiangsu, and Zhejiang provinces [2]. The expanding geographic range of *H. longicornis*—now documented in 441 counties—has been exacerbated by climate warming and increased human encroachment into tick habitats through recreational activities such as hiking and camping [3,4]. Concurrently, livestock infestation rates have risen and the distributions have expanded in rural areas, threatening agricultural productivity and amplifying zoonotic transmission risks [5].

Current tick control strategies heavily rely on chemical acaricides (e.g., cypermethrin) and vegetation management. However, these methods are increasingly challenged by widespread pesticide resistance and environmental contamination [6,7]. For instance, resistance ratios to pyrethroids have reached more than 7× in *H. longicornis* populations in South Korea, drastically reducing efficacy [6]. Integrated pest management (IPM) approaches, though promising, remain underutilized in China due to logistical complexities and limited public awareness [8,9]. Moreover, personal protective measures, such as protective clothing, are impractical for communities in endemic regions, where socioeconomic factors limit access to preventative tools.

Tick repellents represent a critical yet underexplored solution to mitigate human–tick contact. DEET (N,N-diethyl-meta-toluamide), the “gold standard” repellent, has demonstrated >90% efficacy against *Amblyomma americanum* and *Ixodes scapularis* ticks in controlled studies [10]. However, the performance of commercial DEET formulations against *H. longicornis*—especially across its larval, nymphal, and adult stages—remains poorly characterized in China. This study evaluates four DEET-based (5–10% DEET concentrations) and four biont-based repellents available on the Chinese market to determine their hourly repellency rates against *H. longicornis* under controlled laboratory conditions. Our goal is to identify accessible, cost-effective products that balance efficacy with user safety, addressing gaps in practical tick bite prevention strategies for at-risk populations.

## 2. Materials and Methods

### 2.1. Tick Maintenance

Unfed *H. longicornis* (15–60 days old) were obtained from a laboratory colony maintained under controlled conditions with some modifications (27 ± 1 °C, >80% relative humidity, 12:12 light–dark cycle) [11]. Ticks were reared on Kunming mice using a feeding apparatus glued to the shaved dorsum. Ethical approval for animal use was obtained from the Institutional Animal Care and Use Committee (permit number: IACUC1121052600075, date: 26 May 2021).

### 2.2. Test Compounds

In September 2024, we purchased several products for mosquito repellents. Four widely available DEET-based repellents and four commonly used biont-derived repellents were included in this study (Table 1). The concentrations of DEET varied from 5 to 10 percent, which represented the range of commonly purchased repellents in China. The biological repellents were made up of plant- and animal-based extracts, including geranium extract, clove leaf oil, snake bile extract, citronella grass oil, East Indian lemongrass oil, and lemongrass oil (Table 1).

### 2.3. Testing Methods

**Figure 1:**
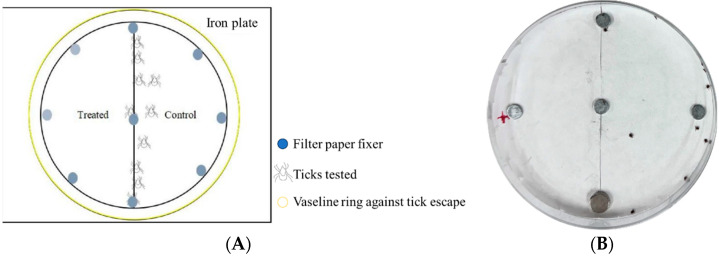
*Haemaphysalis longicornis* evaluated in filter papers impregnated with the repellent commodities. (**A**), Schematic diagram for the evaluation of tick repellent; (**B**), ticks were introduced in filter papers. The left side represented filter paper treated with the repellent, the right side with absolute alcohol. Obviously, ticks aggregated on the right side. (**B**), photo by Weiqing Zheng.

The repellent activity was determined using the previous choice assay with some modification [12,13]. In the experimental group, 9 cm diameter filter paper was bisected into two halves. One half was soaked with 250 μL of the commercial repellent being tested, while the other half was treated with 250 μL of 100% ethanol. A pipette was used to measure and transfer the respective liquids to soak each half of the filter paper, with three replications per group. Simultaneously, a negative control group and a positive control group were established: the negative control used 250 μL of 100% ethanol in place of the commercial repellent, and the positive control used 250 μL of 10% DEET instead of the commercial repellent. After air-drying the filter papers, they were placed in the lid of a Petri dish. The dish lid was aligned with an iron plate and the filter papers were secured to the paper using magnets. Finally, a ring of medical-grade Vaseline was applied to the edge of the Petri dish lid to prevent ticks from escaping. Ticks (20 larvae, 10 nymphs, or 5 adults) were placed in the central position between the treated and control filter papers to initiate the repellency test. The experiment lasted for 1 h, with data recorded every 10 min. For CaliforniaBaby repellent testing, the experiment was conducted over a 6 h period. Observations were performed not only during the first hour (as per the standard protocol for the commercial repellents used in mosquito repellent evaluation), but also at the 2nd, 4th, and 6th hours. After each observation, the ticks were carefully returned to the central position to reset the timer (Figure 1).

### 2.4. Data Treatment and Statistical Analysis

Percent repellency was calculated using the given formula:

repellency (%) = (C − T)/C × 100%.

where C represents the number of ticks climbing on the negative control and T represents the number of ticks climbing on the tested repellent [13,14].

The adjusted repellency was calculated with the below formula:

Ra = (Rn − Rc)/(1 − Rc).

where Ra is the adjusted repellency of a product, Rn represents the repellency of the product, and Rc is the repellency of the negative control set.

Unless indicated, all experiments in this study were performed with three biological replicates. The results are presented as mean values ± standard deviation. The statistical analysis was carried out with Chi square test.

## 3. Results

### 3.1. The Repellency of the Four Commercial DEET-Solved Mosquito Repellents to H. longicornis Ticks

**Figure 2:**
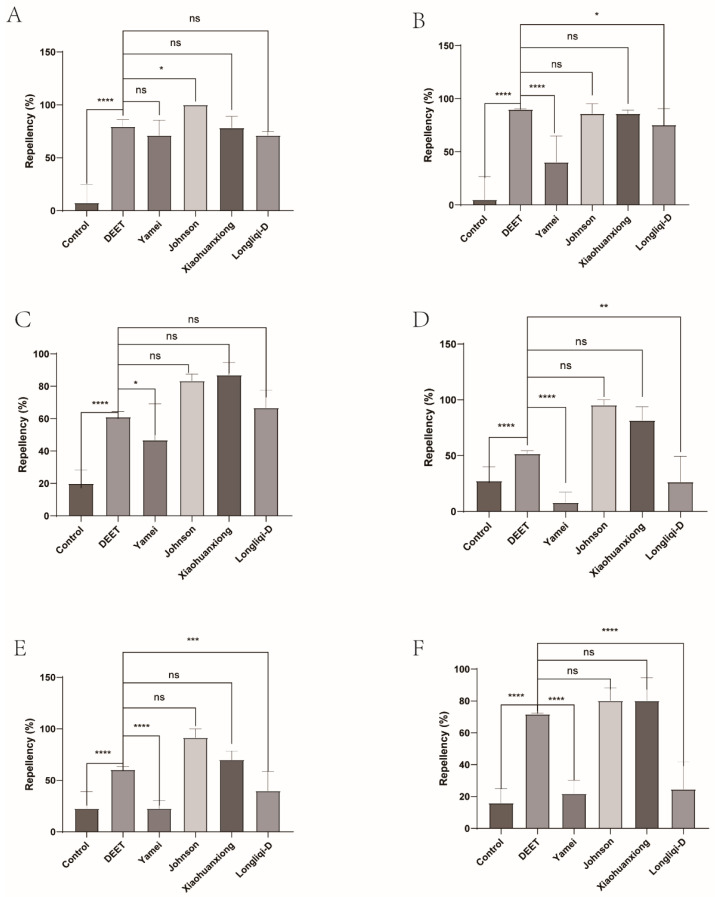
The effect of four commercial DEET-solved mosquito repellents against *Haemaphysalis longicornis* larvae. Note: (**A**), repellency at 10 min post-treatment; (**B**), repellency at 20 min post-treatment; (**C**), repellency at 30 min post-treatment; (**D**), repellency at 40 min post-treatment; (**E**), repellency at 50 min post-treatment; (**F**), repellency at 60 min post-treatment. ns, not significant; * *p* < 0.05; ** *p* < 0.01; *** *p* < 0.001; **** *p* < 0.0001.

**Figure 3:**
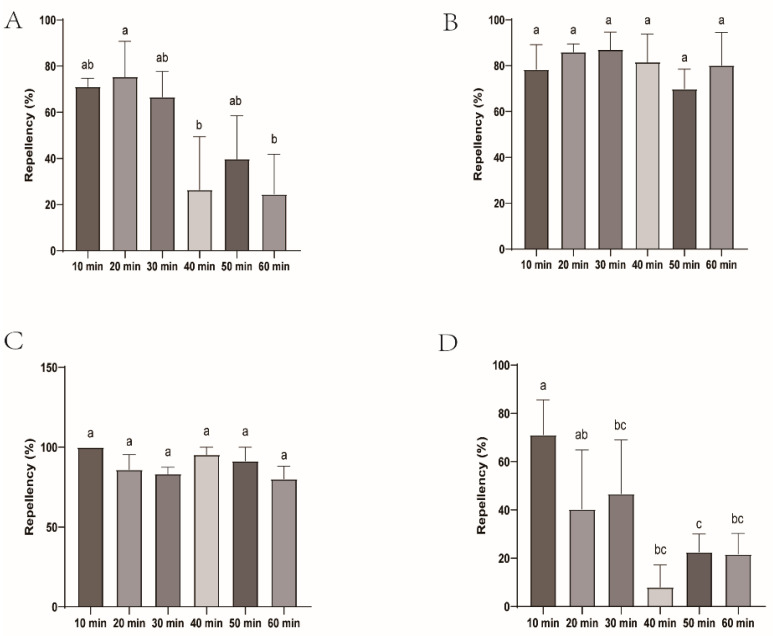
Repellency of *Haemaphysalis longicornis* larvae over time after exposure to four commercial DEET-solved mosquito repellents. Note: (**A**), Longliqi-D; (**B**), Xiaohuanxiong; (**C**), Johnson; (**D**), Yamei. Different letters above the bars in the histogram indicate statistically significant differences.

Johnson emerges as a standout contender, demonstrating near-identical or superior repellency (80.14–100%) against the tick larvae to DEET across multiple intervals. At 10 min, it achieved 100% repellency, statistically surpassing DEET’s 86.04% (*p* = 0.0198), and this superiority maintained all the test process except at 20 min. Xiaohuanxiong also showed strong repellent, and its repellency for the tick larvae remained about 80% for 60 min, except for 69.89% at 50 min. This contrasts with Yamei and Longliqi, which suffered rapid efficacy decay. Yamei’s repellency dropped from 71.17% at 10 min to a dismal 8.05% by 60 min (*p* < 0.0001). Longliqi’s decline was equally stark, collapsing from 75.44% at 20 min to 24.56% by 60 min (*p* < 0.0001), aligning its performance closer to ineffective formulations. We also found environmental factors (e.g., temperature, humidity) affecting baseline tick behavior and then modified the repellency of mosquito repellents by the subtraction of that of the negative control (Figure 2 and Figure 3).

**Figure 4:**
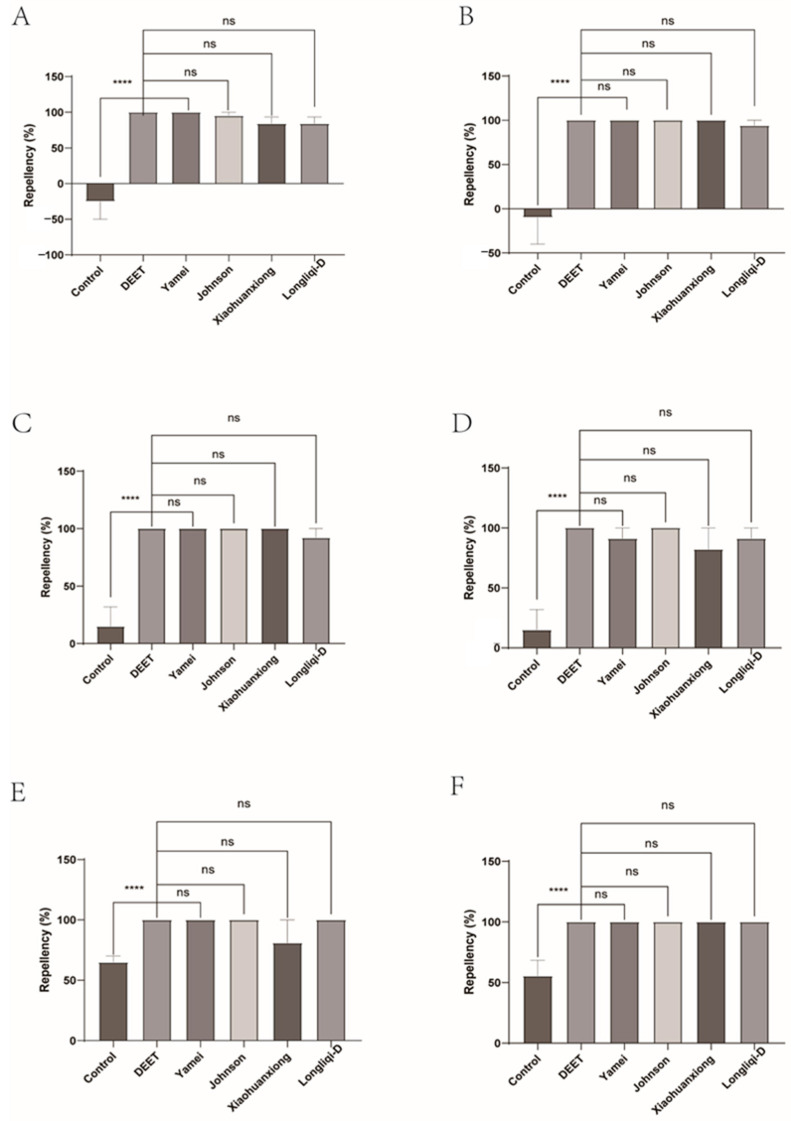
The effect of four commercial DEET-solved mosquito repellents against *Haemaphysalis longicornis* nymphs. (**A**), repellency at 10 min post-treatment; (**B**), repellency at 20 min post-treatment; (**C**), repellency at 30 min post-treatment; (**D**), repellency at 40 min post-treatment; (**E**), repellency at 50 min post-treatment; (**F**), repellency at 60 min post-treatment. ns, not significant; **** *p* < 0.0001.

**Figure 5:**
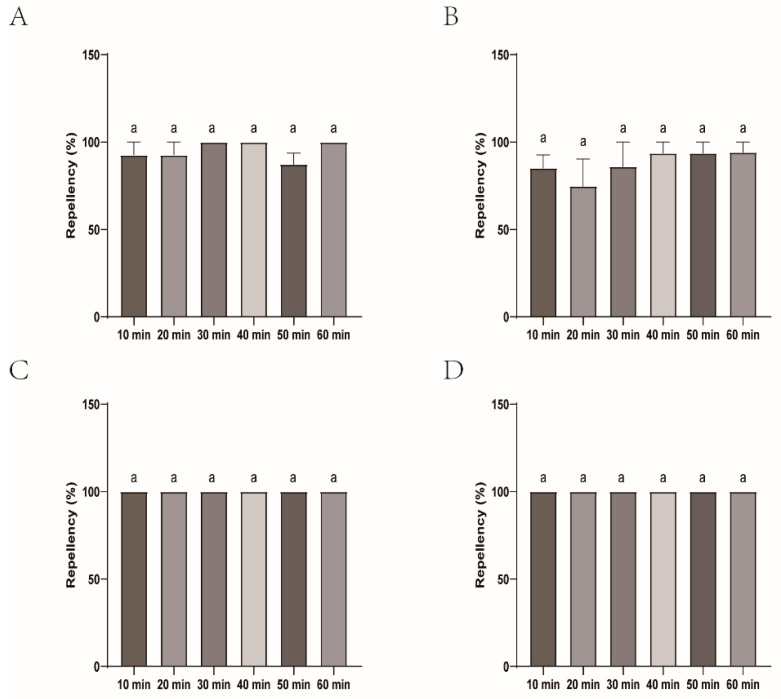
Repellency of *Haemaphysalis longicornis* nymphs over time after exposure to four commercial DEET-solved mosquito repellents. (**A**), Longliqi-D; (**B**), Xiaohuanxiong; (**C**), Johnson; (**D**), Yamei. Bars sharing the same lowercase letter a above the histogram indicate no statistically significant differences between groups.

Regarding *H. longicornis* nymph repellency, all the mosquito repellents had a comparable efficacy with DEET over time. Of them, Johnson exhibited near-perfect immediate and sustained repellency. At 10 min, 94.83% of ticks repelled with borderline significance (*p* = 0.437). By 20 min, it matched DEET with 100% repellency, maintaining this efficacy through 60 min. Yamei was near-perfect with minor declines. It has 100% repellency at most intervals except for 91.11%, with a slight drop at 40 min. Xiaohuanxiong and Longliqi showed subtle variability in repellency, ranging from 80.95% to 100% (Figure 4 and Figure 5).

**Figure 6:**
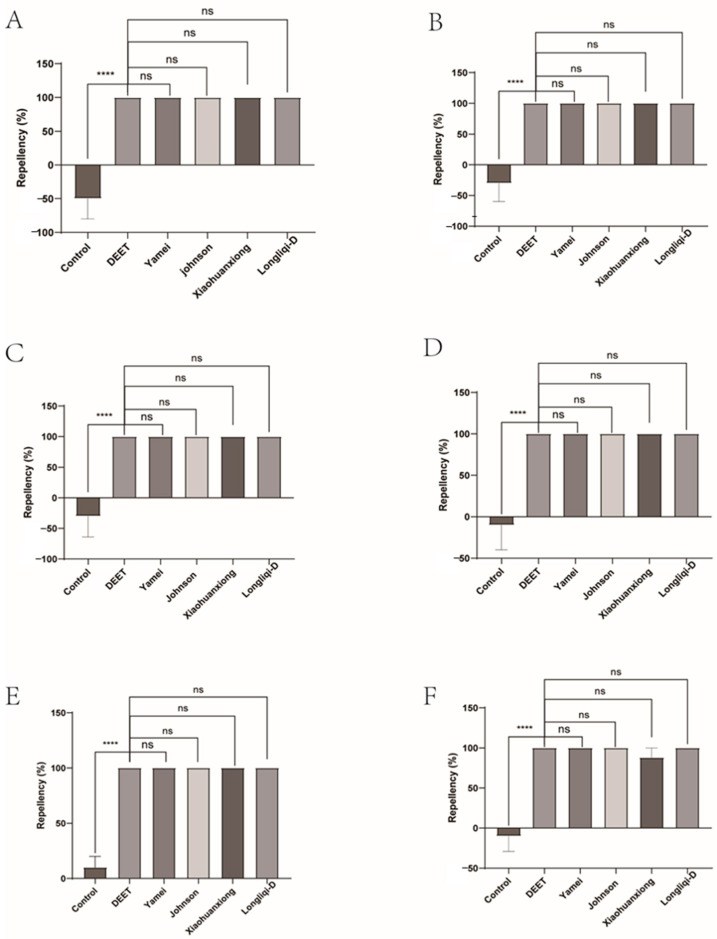
The effect of four commercial DEET-solved mosquito repellents against *Haemaphysalis longicornis* females. (**A**), Repellency at 10 min post-treatment; (**B**), repellency at 20 min post-treatment; (**C**), repellency at 30 min post-treatment; (**D**), repellency at 40 min post-treatment; (**E**), repellency at 50 min post-treatment; (**F**), repellency at 60 min post-treatment. ns, not significant; **** *p* < 0.0001.

**Figure 7:**
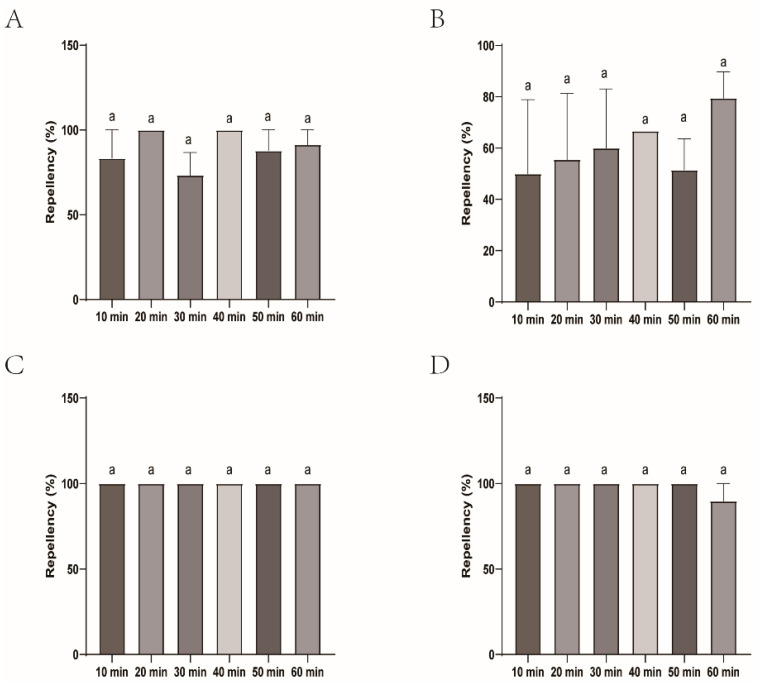
Repellency of *Haemaphysalis longicornis* females over time after exposure to four commercial DEET-solved mosquito repellents. (**A**), Longliqi-D; (**B**), Xiaohuanxiong; (**C**), Johnson; (**D**), Yamei. Bars marked with the same letter a above the histogram indicate no significant differences between them.

The repellency of the female submitted to four commercial DEET-solved mosquito repellents was similar with DEET and, therefore, presented the better efficacy. Johnson, Longliqi, and Yamei were the top performers and demonstrated complete repellency at all intervals (10–60 min). Xiaohuanxiong was also strong, but slightly less stable, and had a minor dip at 60 min with the repellency of 87.88% (Figure 6 and Figure 7).

### 3.2. The Repellency of the Four Commercial Biont-Derived Mosquito Repellents to H. longicornis Ticks

**Figure 8:**
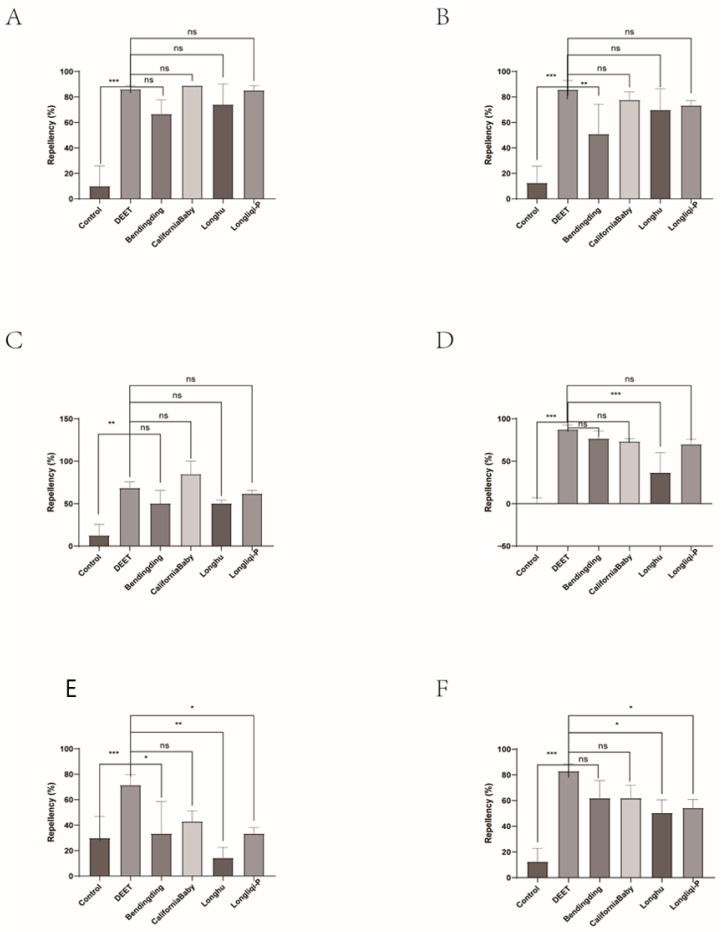
The effect of the four commercial biont-based mosquito repellents against *Haemaphysalis longicornis* larvae. (**A**), Repellency at 10 min post-treatment; (**B**), repellency at 20 min post-treatment; (**C**), repellency at 30 min post-treatment; (**D**), repellency at 40 min post-treatment; (**E**), repellency at 50 min post-treatment; (**F**), repellency at 60 min post-treatment. ns, not significant; * *p* < 0.05; ** *p* < 0.01; *** *p* < 0.001.

**Figure 9:**
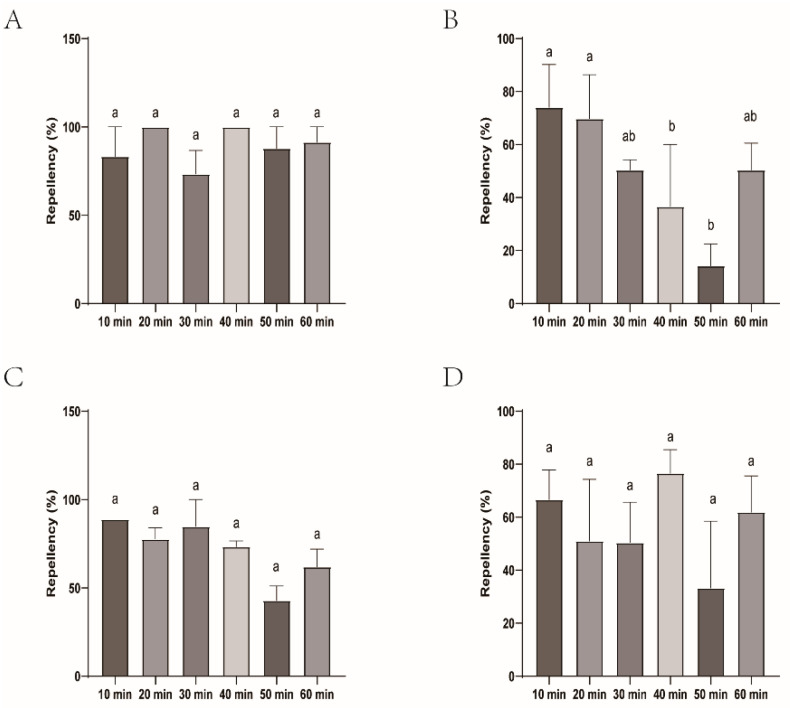
Repellency of *Haemaphysalis longicornis* larvae over time after exposure to the four commercial biont-based mosquito repellents. (**A**), Longliqi-P; (**B**), Longhu; (**C**), CaliforniaBaby; (**D**), Bendingding. Different letters above the bars in the histogram indicate statistically significant differences.

Among the four biont-derived repellents tested, the repellent efficacy against tick larvae varied greatly (14.29–88.89%). CaliforniaBaby demonstrated the highest repellent activity, ranging from 42.86% to 88.89%, characterized by both strong initial efficacy and long-lasting durability. Over a 1 h observation period (evaluated at 10 min intervals), its repellency rate showed no statistically significant difference compared to DEET. Longliqi, Longhu, and Bendingding, however, exhibited unstable repellent activity. All three showed declining efficacy after 20 min. Bendingding and Longliqi displayed gradual reductions in repellency, but no significant differences were observed across the time points. In contrast, Longhu experienced a significant decline in repellent efficacy, changing from 14.29% to 74.07% with statistically significant differences (*p* < 0.05) compared to DEET at 40, 50, and 60 min (Figure 8 and Figure 9).

**Figure 10:**
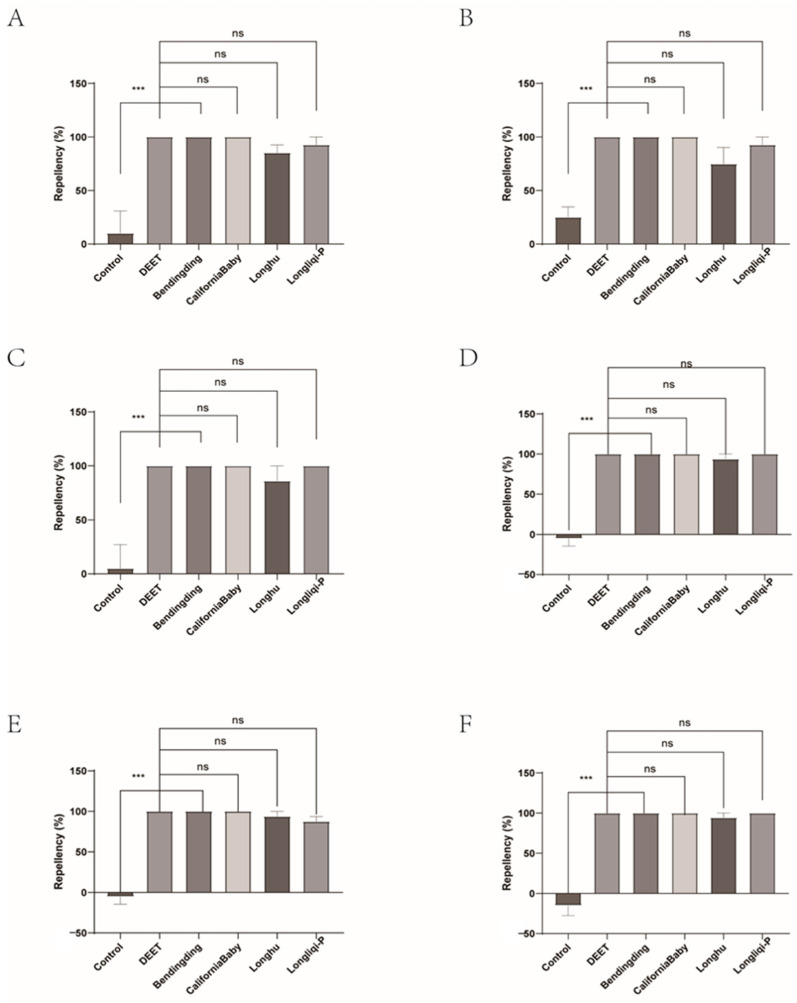
The effect of the four commercial biont-based mosquito repellents against *Haemaphysalis longicornis* nymphs. (**A**), Repellency at 10 min post-treatment; (**B**), repellency at 20 min post-treatment; (**C**), repellency at 30 min post-treatment; (**D**), repellency at 40 min post-treatment; (**E**), repellency at 50 min post-treatment; (**F**), repellency at 60 min post-treatment. ns, not significant; *** *p* < 0.001.

**Figure 11:**
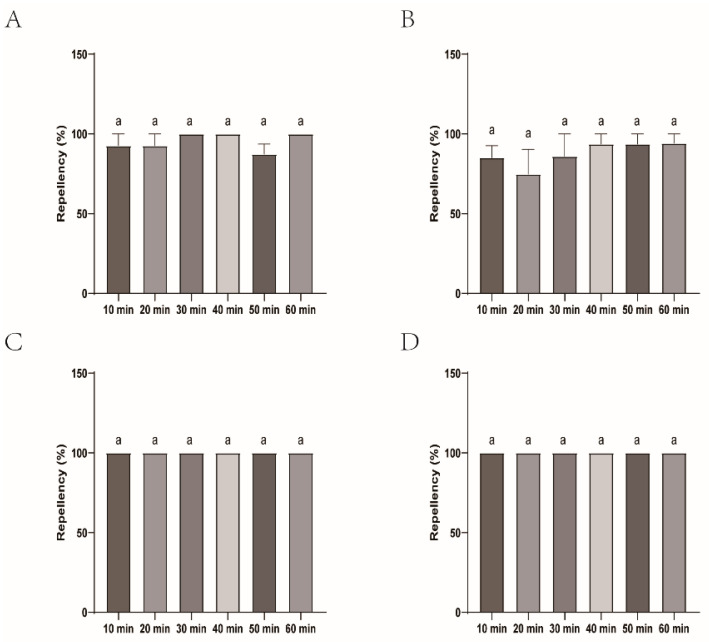
Repellency of *Haemaphysalis longicornis* nymphs over time after exposure to the four commercial biont-based mosquito repellents. (**A**), Longliqi-P; (**B**), Longhu; (**C**), CaliforniaBaby; (**D**), Bendingding. The identical letter a above the bars in the histogram indicate no statistically significant differences.

**Figure 12:**
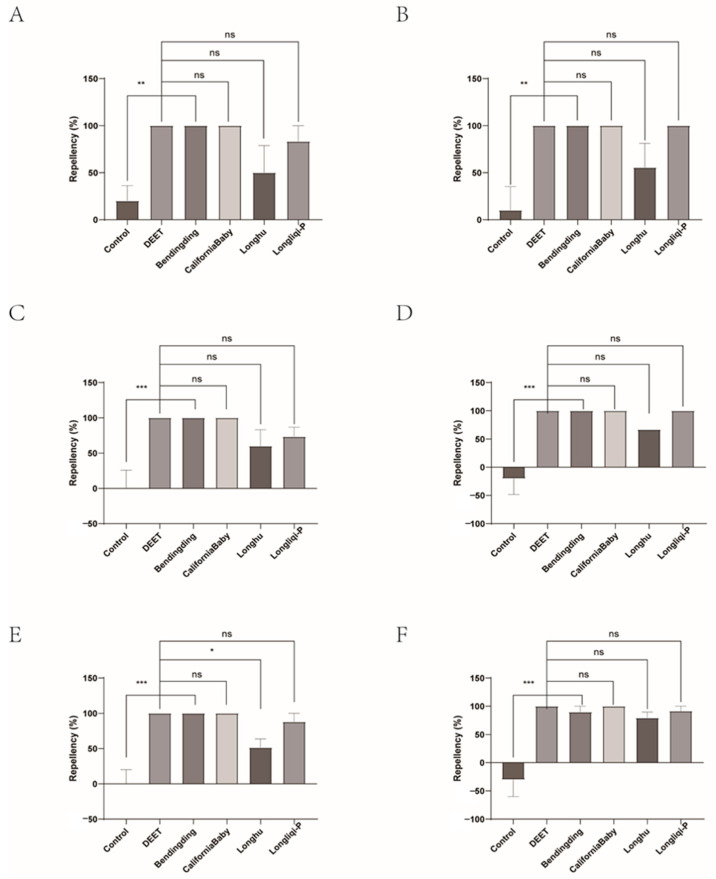
The effect of the four commercial biont-based mosquito repellents against *Haemaphysalis longicornis* females. (**A**), Repellency at 10 min post-treatment; (**B**), repellency at 20 min post-treatment; (**C**), repellency at 30 min post-treatment; (**D**), repellency at 40 min post-treatment; (**E**), repellency at 50 min post-treatment; (**F**), repellency at 60 min post-treatment. ns, not significant; * *p* < 0.05; ** *p* < 0.01; *** *p* < 0.001.

**Figure 13:**
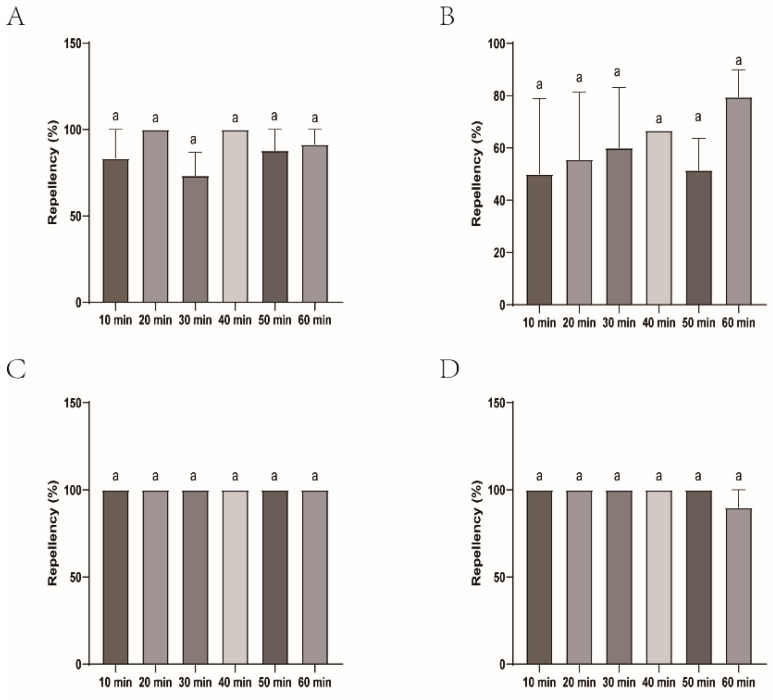
Repellency of *Haemaphysalis longicornis* females over time after exposure to the four commercial biont-based mosquito repellents. (**A**), Longliqi-P; (**B**), Longhu; (**C**), CaliforniaBaby; (**D**), Bendingding. The same letter a above the bars in the histogram indicate no statistically significant differences.

During repellency testing against nymphal and adult ticks, CaliforniaBaby and Bendingding demonstrated complete efficacy and sustained prolonged activity, except for the 89.74% repellency of Bendingding against adult ticks at 60 min. Longliqi-P also showed robust repellency to nymphs and adults with repellent rates ranging from 73.33% to 100%; however, its efficacies of nymphs (77.78–100%) were better than those of adults (73.33–100%). Longhu had the worst repellency (57.89–100%) for nymphs and the worst repellency (50–79.49%) against adults among four commercial biont-based mosquito repellents. It exhibited a statistically significant decrease in repellency against adult ticks at 50 min compared to DEET (*p* < 0.05). Among the four biont-derived repellents, Bendingding and CaliforniaBaby consistently displayed the highest efficacy and temporal stability, maintaining a strong repellent performance with minimal decline over the evaluation period (Figure 10, Figure 11, Figure 12 and Figure 13).

### 3.3. Repellent Performance of CaliforniaBaby at Intervals

CaliforniaBaby, a biont-derived insect repellent, now has gained significant market acceptance and showed good tick-repellent efficacy and sustained residual activity. To further evaluate its potential as a viable tick repellent, we extended testing durations to assess its repellent performance at 2, 4, and 6 h intervals. Repellency assays demonstrated that CaliforniaBaby achieved comparable efficacy to DEET, maintaining equivalent repellency rates over the 6 h period, namely 76.92%, 76.13%, and 84.62%. These findings position CaliforniaBaby as a promising DEET-free alternative for long-lasting tick protection (Table 2).

## 4. Discussion and Conclusions

The experimental results across larval, nymphal, and adult developmental stages clearly demonstrate varying sensitivities to repellents within the same tick species. Notably, nymphs exhibited the highest sensitivity to repellents. Similar conclusions were reported by Kulma et al. (2019) who observed that *Ixodes ricinus* nymphs showed significantly greater sensitivity to DEET than adult females in in vitro assays [15].

In larval experiments, partial “swaying” gait patterns were observed in the DEET-positive control group after larvae entered DEET-treated zones. This aligns with findings by Koloski et al. (2020) who documented uncoordinated movement and convulsive behaviors in *Dermacentor variabilis* exposed to DEET [16]. Further evidence from Koloski et al. (2019) indicated a rapid, marked reduction in acetylcholinesterase gene transcription levels in DEET-exposed *D. variabilis*, suggesting a potential mechanism underlying the observed gait abnormalities [17].

This study evaluated the repellent efficacy of four commercial DEET-based mosquito repellents and four biont-derived formulations against ticks. All products demonstrated strong repellent activity, particularly against nymphs and adults, with a performance comparable to DEET. These findings suggest that mosquito repellents may also serve as effective tick repellents for nymphs and adults, though their efficacy against larvae remains suboptimal. Therefore, assessing larval repellency is critical for achieving robust field protection. Interestingly, DEET efficacy did not strictly correlate with the concentration; for example, Johnson’s 7% DEET formulation outperformed Yamei’s 10% DEET product. This discrepancy may stem from differences in manufacturing processes or auxiliary ingredients, emphasizing the importance of optimizing production techniques and formulations to enhance the active ingredient performance.

Biont-derived repellents, valued for their environmental compatibility and low human toxicity, are a vital complement to synthetic alternatives. The market analysis of purported biont-based products revealed that CaliforniaBaby exhibited nymphal and adult tick repellency equivalent to DEET over 6 h. CaliforniaBaby repellent contains extracts of citronella grass and East Indian lemongrass, which have been proven effective against various tick species. Citronella oil exhibits strong repellency, with studies showing a 92.4% efficacy at 10 μg/cm^2^ against *A. americanum* nymphs [14], while a 10% formulation maintained efficacy against *I. ricinus* nymphs for up to 8 h (89–90% repellency) [18]. Against *Dermacentor reticulatus* adults, 1% citronella oil achieved 82.0% repellency [19]. However, 10% citronella oil provided less than 30 min of protection against *I. scapularis* female ticks [20]. Lemongrass oil (*Cymbopogon flexuosus*) displayed dose-dependent activity, with repellency rising from 30% to 90% at concentrations of 7–56 μL/mL against *Rhipicephalus sanguineus* sensu lato adults, maintaining full efficacy for 1 h at the highest dose [21]. When tested on *I. scapularis* nymphs, lemongrass oil exhibited a time-dependent decline in repellency, dropping from 83.0% (3 min post-treatment) to 68.8% after 10 min at 57.5 μg/μL. The primary active compounds, geraniol and citral, demonstrated superior efficacy and persistence compared to crude lemongrass oil. A Canadian commercial geraniol-based repellent achieved 100% efficacy after 5 min, though this decreased to 62.5% by 2 h [22]. Notably, 10% geraniol provided 1 h of protection against *I. scapularis* female ticks [20], highlighting its enhanced performance over citronella-based formulations.

A critical observation is that a product labeled as “biont-derived” in the present study was found to contain DEET as the primary active ingredient, lacking detectable bioactive phytochemicals. Other remaining products were free of synthetic additives and biont-derived ingredients were fully consistent with the trademark labeling. They contained repellent-active biont metabolites including geranium extract and clove leaf oil. Geranium essential oil demonstrated significant repellency against *A. americanum* nymphs at 50 μg/cm^2^, with an efficacy of 89.5%, which was markedly higher than the negative control [14]. In contrast, a 10% geranium oil formulation showed no observable repellent effect against nymphal *I. ricinus* ticks [18]. Clove ethanolic extract exhibited concentration-dependent repellency against *Rhipicephalus microplus* larvae, characterized by an initially rapid increase followed by a plateau effect at higher concentrations. At 100 mg/mL, its repellency was 86.48% at 0 h, decreasing to 26.9% after 48 h. The median effective concentration (EC₅₀) at 0 h was determined to be 56.20 mg/mL [23]. Similarly, clove essential oil (50 μg/cm^2^) displayed a high repellency rate of 91.3% against *A. americanum* nymphs, significantly outperforming the control group [14]. A 10% clove oil formulation exhibited time-dependent activity against *I. ricinus* nymphs, maintaining repellency rates of 68%, 82%, and 78% at 4, 6, and 8 h post-treatment, respectively [18]. Moreover, the topical application of 10% clove oil provided human protection against *I. scapularis* female ticks for up to 1.5 h [20].

This study demonstrates that commercially available DEET-based and select biont-derived mosquito repellents exhibit significant repellent activity against *H. longicornis*, supporting their repurposing for tick bite prevention. The key findings reveal that ticks showed the high repellent sensitivity to DEET-based repellents, with Johnson’s 7% DEET achieving near-complete repellency. Some of biont-derived mosquito repellents also demonstrated good repellency to ticks and CaliforniaBaby matched DEET’s 6 h repellency, indicating its utility in prolonged protection against ticks.

## Figures and Tables

**Table 1:** Widely available mosquito repellents tested in this study.

No.	Product	Component	Registration Number
1	Yamei	10%DEET	WP20100128
2	Longliqi-D	5%DEET	WP20080377
3	Johnson	7%DEET	WP20110021
4	Xiaohuanxiong	5%DEET	WP20110008
5	Longhu	Geranium extract and clove leaf oil	Huzhuang20160147
6	Longliqi-P	Snake bile extract	Suzhuang20190030
7	CaliforniaBaby	Extracts of citronella grass and East Indian lemongrass	1051183-30002-AA
8	Bendingding	Lemongrass essential oil	Unavailable

**Table 2:** Repellency index (±SE) of CaliforniaBaby after different drying times in hours in unfed *Haemaphysalis longicornis* nymphs.

Product	Time (h)	Repellency (%)	Classification
Control	2	13.33 ± 13.33	Neutral
	4	3.70 ± 9.94	Neutral
	6	12 ± 5.95	Neutral
DEET	2	76.92 ± 13.32	Repellent
	4	84.62 ± 6.92	Repellent
	6	92.31 ± 7.58	Repellent
CaliforniaBaby	2	76.92 ± 13.32	Repellent
	4	76.13 ± 11.99	Repellent
	6	84.62 ± 7.58	Repellent

Note: Ten nymphs were employed in the repellency studies for each replication of the control, DEET and CaliforniaBaby groups. Each group was carried out with three replications.

## Data Availability

The original contributions presented in this study are included in the article. Further inquiries can be directed to the corresponding authors.
